# Цефалгический синдром у больных акромегалией

**DOI:** 10.14341/probl13423

**Published:** 2024-11-04

**Authors:** Г. М. Нуруллина, И. Н. Пушкарев, Е. Г. Пржиялковская

**Affiliations:** Первая республиканская клиническая больница; Ижевская государственная медицинская академия; Научный медицинский исследовательский центр эндокринологии

**Keywords:** акромегалия, аденома гипофиза, головная боль

## Abstract

Целью данного обзора является обобщение имеющихся в литературе данных о причинах головной боли, возникающей у пациентов с акромегалией, а также о влиянии различных методов лечения акромегалии на головную боль. Поиск публикаций производился в базе данных PubMed по ключевым словам: «Headache in patients with acromegaly», «Headache in patients with pituitary adenomas», «Tension-type headache», «Migraine». Головная боль у пациентов с аденомами гипофиза, секретирующими соматотропный гормон (СТГ), — нередкое явление: по данным разных авторов, цефалгический синдром встречается у 30–70% больных акромегалией и может ухудшать качество их жизни наряду с другими факторами, вплоть до инвалидизации. По характеру развития головная боль при акромегалии классифицируется на первичную (мигрень, головная боль напряжения, тригеминальные вегетативные цефалгии, например, SUNCT-синдром и кластерные головные боли), а также может вторично вызываться различными причинами, связанными непосредственно с опухолью. Все это требует дифференциальной диагностики. Факторы, вызывающие головные боли при соматотропиномах, пока недостаточно хорошо изучены и требуют дальнейших исследований. К ним относят масс-эффект опухоли, гормональную гиперсекрецию, патологию височно-нижнечелюстного сустава, задержку в организме натрия и жидкости, психологические факторы и др. Авторами проводилась оценка влияния на головную боль различных методов лечения акромегалии: трансназальной транссфеноидальной аденомэктомии, лучевой терапии и медикаментозной терапии аналогами соматостатина, агонистами дофамина и антагонистом рецепторов гормона роста. Однако даже при достижении нормальных уровней СТГ и инсулиноподобного фактора роста 1 (ИФР-1) цефалгический синдром может сохраняться, поэтому пациенты должны быть об этом предупреждены и направлены к цефалгологу для подбора адекватной терапии головной боли.

## Abstract (en)

The aim of this review is to summarize the data available in the literature on the causes of headache in patients with acromegaly, as well as on the effect of various methods of acromegaly treatment on headache. Publications were searched in the PubMed database using the keywords «Headache in patients with acromegaly», «Headache in patients with pituitary adenomas», «Tension-type headache», «Migraine». Headache in patients with pituitary adenomas secreting somatotropic hormone (STH) is not uncommon: according to various authors, cephalgic syndrome occurs in 30–70% of patients with acromegaly and can worsen their quality of life, along with other factors, up to disability. By the nature of development, headache with acromegaly is classified into primary (migraine, tension headache, trigeminal autonomic cephalgia, for example, SUNCT syndrome and cluster headaches), and can also be caused by various causes directly related to the tumor. All this requires differential diagnosis. The factors causing headaches in somatotropinomas have not yet been well studied and require further research. These include the mass effect of the tumor, hormonal hypersecretion, pathology of the temporomandibular joint, sodium and fluid retention in the body, psychological factors, etc. The authors evaluated the effect on headache of various methods of acromegaly treatment: transnasal transsphenoidal adenomectomy, radiation therapy and drug therapy with somatostatin analogues, dopamine agonists and growth hormone receptor antagonist. However, even when normal levels of STH and insulin-like growth factor 1 (IGF-1) are reached, cephalgic syndrome may persist, therefore patients should be warned about this in advance and referred to a cephalgologist to select adequate headache therapy.

## ВВЕДЕНИЕ

Гипофиз — главная эндокринная железа, расположенная в пределах турецкого седла клиновидной кости. По бокам от турецкого седла расположены кавернозные синусы, заполненные III, IV, V (глазной и верхнечелюстной нервы) и VI парами черепно-мозговых нервов, а также внутренней сонной артерией (ВСА). Сверху турецкое седло покрыто твердой мозговой оболочкой — диафрагмой, над которой находится перекрест зрительных нервов — хиазма. Спереди гипофиз граничит с твердой мозговой оболочкой, покрывающей заднюю стенку пазухи клиновидной кости [1]. При увеличении размеров гипофиза за счет опухоли данные внутричерепные структуры могут смещаться, что активирует систему тройничного нерва и ведет к высвобождению ряда нейропептидов, таких, как кальцитонин-ген-родственный пептид (CGRP) и пептид, активирующий аденилатциклазу гипофиза (PACAP), играющих одну из ключевых ролей в возникновении головной боли, в частности мигрени [2].

Аденома гипофиза — доброкачественная опухоль, наиболее распространенная патология этого органа. Около 25–30% аденом являются гормонально-неактивными [3], остальные же выделяют избыток биологически активного гормона в зависимости от клеточного состава [4]. Наиболее распространены пролактиномы (до 60% всех аденом), секретирующие пролактин; на долю соматотропином приходится около 20%; кортикотропиномы встречаются в 1–2% случаев; тиреотропиномы, гонадотропиномы аденомы, а также смешанные клеточные аденомы являются редкими. До 80–90% всех гормонально-неактивных опухолей составляют гонадотропиномы [1][4].

Несмотря на то, что многие аденомы обнаруживаются случайно в ходе нейровизуализации, они могут сопровождаться целым рядом офтальмологических (снижение остроты зрения, сужение полей зрения, частичная атрофия зрительного нерва и др.), неврологических и/или эндокринных проявлений. При акромегалии головная боль может быть достаточно выраженным и даже инвалидизирующим симптомом, о котором сообщают, по разным данным, от 30 до 70% пациентов и более [5][6]. Наиболее распространенным типом головной боли при акромегалии является мигрень (по данным разных авторов, встречается у 30–50% больных, отмечающих у себя головные боли), далее следует головная боль напряжения (ГБН), еще реже отмечаются тригеминальные вегетативные цефалгии (TACs), к которым относятся кластерные головные боли, кратковременная односторонняя невралгическая головная боль с инъецированием конъюнктивы и слезотечением (SUNCT-синдром) и др. [7–10]. Тем не менее патофизиологические механизмы, лежащие в основе головной боли при акромегалии, еще не до конца изучены и требуют дальнейших исследований [11].

## ПАТОГЕНЕЗ

Развитие головной боли при акромегалии объясняется различными механизмами. Возможными причинами могут являться масс-эффект опухоли, который наблюдается у пациентов с опухолями больших размеров (инвазия в кавернозный синус, смещение внутричерепных структур, чувствительных к боли, таких как кровеносные сосуды, черепно-мозговые нервы и твердая мозговая оболочка, интраселлярная гипертензия), гиперсекреция гормона роста, гиперпролактинемия, задержка натрия и жидкости в организме, патология височно-нижнечелюстного сустава (ВНЧС), апоплексия гипофиза, а также психологические факторы [11–13].

## Масс-эффект опухоли

Ранее в качестве объяснения головных болей, возникающих при аденомах гипофиза, предлагалось распространение опухоли за пределы турецкого седла, что влечет за собой растяжение твердой мозговой оболочки [14]. Тем не менее у некоторых пациентов с акромегалией даже при эндоселлярных микро- и макроаденомах головная боль является основной жалобой. Кроме того, некоторая часть пациентов с аденомами гипофиза больших размеров и сильной головной болью не сообщали об ее уменьшении даже после оперативного лечения [5]. Таким образом, данная причина не может быть основной.

Существуют предположения [14], что головные боли могут возникать вследствие инвазии опухоли в кавернозные синусы с поражением первых двух ветвей тройничного нерва (глазного и верхнечелюстного нервов), сдавлением нервного сплетения, окружающего ВСА, а также активацией тройничного нерва через растяжение твердой мозговой оболочки турецкого седла [1][15]. При этом возникает активация тригеминоваскулярной системы, состоящей из оболочек головного мозга, внутричерепных кровеносных сосудов и иннервирующих эти структуры аксонов тройничного ганглия, что в свою очередь ведет к высвобождению CGRP и PACAP, участвующих в развитии головных болей [2]. В своем проспективном исследовании Yu et al. связали инвазию гормонально-неактивных аденом в кавернозный синус с возникновением головных болей у 97 пациентов. Степень инвазии определяли с использованием классификации Knosp, при этом 3-я степень указывает на распространение опухоли латеральнее по отношению к латеральной касательной к ВСА, а 4-я степень указывает на полное окружение кавернозного сегмента ВСА. Частота головной боли, связанной с гормонально-неактивными аденомами, составила 48,5% (47 пациентов), 70% из которых имели 3 или 4 степень инвазии по Knosp [3][15].

Повышение интраселлярного давления — сдавление гипофиза, увеличенного за счет аденомы костными структурами и твердой мозговой оболочкой внутри турецкого седла, — является еще одним предполагаемым механизмом развития головной боли у пациентов с аденомой гипофиза [1][11][14][15]. Было показано, что увеличение размеров аденомы гипофиза значительно увеличивает интраселлярное давление одновременно с головными болями [11][14]. Исследование Arafah et al., включавшее 49 пациентов, показало, что среднее значение интраселлярного давления было значительно выше у пациентов с головными болями по сравнению с контрольной группой без головных болей [16]. Интраоперационное определение интраселлярного давления в исследовании Hayashi et al. (2019 г.) среди 30 пациентов также показало похожие результаты. После транссфеноидальной аденомэктомии снижение интраселлярного давления было больше у пациентов с головной болью, а у 28 из 30 пациентов (93,3%) головная боль значительно облегчилась [17].

## Гормональная гиперсекреция

Небольшие гормонально-активные опухоли могут сопровождаться сильными головными болями без инвазии в кавернозный синус или растяжения твердой мозговой оболочки [15]. Это указывает на то, что непосредственно гиперсекреция соматотропного гормона, вероятно, может играть большую роль в возникновении головных болей и их усилении, независимо от размера аденомы. Существуют предположения о том, что соматотропиномы и пролактиномы сами по себе могут опосредовать возникновение боли (быть проноцицептивными) [11]. Это подтверждается облегчением головной боли на фоне лечения аналогами соматостатина, даже в отсутствие уменьшения размера опухоли [15]. На основе продемонстрированного октреотидом анальгезирующего эффекта было выдвинуто предположение, что аналоги соматостатина могут ингибировать некоторый, пока не обнаруженный, проноцицептивный пептид, вызывающий головную боль у пациентов с акромегалией [18]. Еще одним аргументом в пользу возможной роли соматотропного гормона в механизме развития головной боли являются данные, полученные во время исследования заместительной терапии гормоном роста у детей с его дефицитом. В когорте из 57 968 детей, получавших супрафизиологические дозы рекомбинантного человеческого гормона роста, сообщалось о головной боли с частотой 793,5 на 100 000 пациенто-лет в начале терапии [12].

## Воспаление

Воспаление в гипофизарной области представляет собой еще один патофизиологический механизм, который может объяснять развитие головной боли [11][15]. Считается, что быстрое увеличение гипофиза из-за отека и инфильтрации лимфоцитами приводит к растяжению менингеальных структур и вызывает головную боль [11].

## Нейропептиды

Известна роль различных нейропептидов в возникновении первичных головных болей. Мозговое кровообращение контролируется нервными волокнами, содержащими нейропептид-Y (NPY), вазоактивный интестинальный пептид (VIP), субстанцию P и кальцитонин-ген-родственный пептид (CGRP). Эти нейропептиды опосредуют сокращение (NPY) и дилатацию (VIP, субстанция P, CGRP) кровеносных сосудов [5]. Также известно, что в развитии головной боли играет роль пептид, активирующий аденилатциклазу гипофиза (PACAP) [7][19]. Вероятно, что все эти биохимические факторы при аденомах гипофиза участвуют в патогенезе головной боли [11]. В проспективном исследовании Zhang Y. et al. были исследованы концентрации CGRP и PACAP у 63 пациентов с аденомами гипофиза, 33 (52,4%) из которых отмечали головную боль. Было показано, что в группе пациентов с головной болью уровни CGRP и PACAP в плазме крови были в несколько раз выше, чем у пациентов без головной боли [7].

## Внутричерепная гипертензия

В литературе существует всего два описанных случая головной боли при акромегалии, связанной с внутричерепной гипертензией (ВЧГ) [20][21]. Однако, как уже было сказано выше, имеется множество сообщений о головной боли вследствие ВЧГ, возникающей на фоне лечения рекомбинантным человеческим гормоном роста (ГР) детей с дефицитом соматотропного гормона (СТГ) [12], а также на фоне терапии инсулиноподобным фактором роста 1 типа (ИФР-1) у лиц с редко встречающейся резистентностью к ГР [12, 22]. Данная причинно-следственная связь подтверждалась тем, что симптомы ВЧГ уменьшались после отмены препаратов ГР и возобновлялись после возвращения терапии у некоторых пациентов [22]. Помимо головной боли, ВЧГ может приводить к нарушениям зрения (снижению остроты зрения, диплопии, страбизму, одно- или двустороннему отеку диска зрительного нерва), тошноте и рвоте [12]. Механизм развития ВЧГ остается неясным, однако предполагается, что она может развиваться в результате повышенной выработки ликвора и/или нарушения его оттока [22]. К этому может приводить задержка натрия и внеклеточной жидкости в организме. Lampit et al. обнаружили, что при введении рекомбинантного ГР у детей кратковременно возрастала активность ренин-ангиотензин-альдостероновой системы (РААС), что вело к снижению осмоляльности плазмы крови и повышению осмоляльности мочи [12][23].

## Артериальная гипертензия

Одним из основных и наиболее частых осложнений акромегалии является синдром артериальной гипертензии (АГ), который встречается в среднем в 35% случаев [24]. По сравнению с общей популяцией у пациентов с акромегалией чаще выявляется повышенный уровень диастолического артериального давления (АД), а также среди них более распространено суточное колебание АД по типу non-dipper [25]. Патогенез АГ при акромегалии до сих пор изучается, но уже сейчас предполагается ее мультифакториальный характер. К подъему АД у пациентов с акромегалией могут приводить активация РААС и, как следствие, задержка натрия и воды в организме, которые вызывают периферические отеки, увеличение массы тела, синдром запястного канала [21, 22]; повышение общего периферического сосудистого сопротивления (за счет гипертрофии стенок артерий, эндотелиальной дисфункции, активации СНС) и сердечного выброса, а также синдром обструктивного апноэ сна (СОАС) [25]. Помимо этого, к задержке натрия и воды также могут приводить ингибирование секреции предсердного натрийуретического пептида ИФР-1 и антинатрийуретический эффект инсулина [25].

Как известно, значительное повышение АД может сопровождаться головной болью (повышение систолического давления ≥180 мм рт.ст. и/или диастолического давления ≥120 мм рт.ст.). В международной классификации головной боли 3-го пересмотра имеется рубрика, посвященная головной боли, связанной с АГ [26]. Помимо этого, в новых исследованиях была выявлена связь АГ и мигрени [27][28]. Так, Finocchi et al. описывают общие для мигрени и АГ патогенетические механизмы, такие как эндотелиальная дисфункция, гиперактивация РААС, недостаточная активность вегетативной нервной регуляции [27].

## Патология височно-нижнечелюстного сустава

Акромегалия может являться причиной патологии ВНЧС, которая имеет миогенный и артрогенный компонент. Помимо этого, при заболеваниях ВНЧС может поражаться тройничный нерв [13]. Гиперсекреция СТГ и ИФР-1 приводит к непропорциональному росту нижней и верхней челюстей, ослаблению связок и сухожилий в области ВНЧС, росту остеофитов. У больных акромегалией могут наблюдаться прогнатизм, диастема [29]. Патология ВНЧС сопровождается такими симптомами, как головная и лицевая боли, боль в нижней челюсти, оталгия [30]. По данным литературы, хроническая мигрень встречается в 8 раз чаще у пациентов с патологией ВНЧС, чем в общей популяции [31].

## Апоплексия гипофиза

Апоплексия гипофиза — достаточно редкая клиническая ситуация, связанная с кровоизлиянием в ткань железы либо с ее инфарктом. При этом 80–90% пациентов отмечают возникновение в лобной или ретрообитальной областях сильнейшего цефалгического синдрома, который может сопровождаться тошнотой, рвотой, офтальмологическими нарушениями (снижение остроты зрения, параличи III, IV, VI пар черепно-мозговых нервов, битемпоральной гемианопсией) [32]. Разные авторы указывают распространенность апоплексии гипофиза среди пациентов с аденомами гипофиза от 2 до 12% [33]. Предполагается, что возникновение головной боли связано с повышением интраселлярного давления, а также с растяжением и раздражением менингеальных оболочек, о чем было сказано выше [34].

## Психологические факторы

Психические расстройства, такие как депрессия и тревога, связанные с физическими аспектами заболевания (изменения внешности, болевой синдром различной локализации, проведение оперативного вмешательства, сопутствующая патология и осложнения, инвалидизация и т.д.) и значительно ухудшающие качество жизни пациентов, очень часто развиваются у пациентов с акромегалией [35][36]. По данным крупного проекта Eurolight, проводившегося в Европе, в котором исследовалась первичная головная боль, была выявлена связь между мигренью, депрессивными и тревожными расстройствами. Кроме того, более сильная связь наблюдалась между лекарственно-индуцированной головной болью (ЛИГБ, абузусная головная боль), депрессией и тревогой, а головная боль напряжения была ассоциирована только с тревогой [37].

Нарушения сна также являются важным фактором риска первичных головных болей, особенно головной боли напряжения. Качество сна часто ухудшается у пациентов с заболеваниями гипофиза, особенно при акромегалии [11]. Это может быть связано с СОАС, бруксизмом (который тоже приводит к патологии ВНЧС), бессонницей и другими причинами [38].

## КЛИНИЧЕСКАЯ КАРТИНА

Несмотря на то, что о головной боли сообщают до 70% пациентов с соматотропиномами, ее наличие может быть не связано непосредственно с опухолью, так как головная боль сама по себе является довольно распространенной жалобой в общей популяции. Тем не менее СТГ-секретирующую аденому гипофиза можно связать с головной болью в случае, если боль возникла впервые и сочетается с другими симптомами акромегалии [18][39].

В проспективном исследовании Levy et al. (2005 г.) были проанализированы 84 пациента с опухолью гипофиза (33% — акромегалия) и головной болью. Наиболее распространенными синдромами являлись хроническая мигрень, часто возникающая у пациентов с предшествующей мигренью (46%), и эпизодическая мигрень (30%) [8][18]. Следующей по частоте была первичная колющая головная боль (27%). В гораздо меньшей степени проявлялись тригеминальные вегетативные цефалгии (TACs), к которым относятся кратковременные односторонние невралгические головные боли с инъекцией в конъюнктиву и слезотечением (SUNCT) (5%), кластерная головная боль (4%) и гемикрания континуа (1%). Шесть проявлений головной боли (7%) не могли быть классифицированы в соответствии с критериями Международной классификации головной боли 2-го пересмотра [8].

В еще одном проспективном исследовании, проведенном Schankin et al. (2012 г.), головная боль встречалась у 24 из 58 (41%) пациентов с аденомами гипофиза, причем мигрень была зарегистрирована у 11 пациентов (45,8%), головная боль напряжения — у 8 (33,3%), у 4 (16,7%) — и то и другое, мигрень в сочетании с кластерной головной болью — у 1 (4,2%) [9].

В одномоментном исследовании Dallel et al. (2021 г.), в котором приняли участие 146 пациентов с аденомами гипофиза (19,9% — акромегалия), были получены следующие результаты: испытывали головную боль 79 опрошенных (54,1%), из которых 44 (55,7%) сообщили о мигрени, оставшиеся участники испытывали головную боль других типов [10].

В международной классификации головной боли 3-го пересмотра головная боль при акромегалии классифицируется как головная боль, связанная с избыточностью или недостаточностью секреторной функции гипоталамуса или гипофиза [26]. Ее клинические проявления в целом аналогичны первичной головной боли и несильно отличаются от таковых при других формах гормонально-активных и неактивных аденом гипофиза [5].

Головные боли при акромегалии могут локализоваться в любых областях, но чаще всего описываются как фронтальные. Боли могут быть как двусторонними, так и односторонними [1]. В ретроспективном обзоре Wang et al. сообщалось, что у пациентов и с гормон-продуцирующими, и с гормонально-неактивными аденомами гипофиза головная боль соответствовала стороне опухоли [40]. Yu et al. также отмечали, что при односторонних головных болях, связанных с аденомой гипофиза, инвазия в кавернозный синус была ипсилатеральной по отношению к головной боли [3]. В исследовании Levy et al. корреляция между инвазией в кавернозный синус и ипсилатеральной головной болью была выявлена у 10 из 18 пациентов [8].

## ЛЕЧЕНИЕ

В литературе оценивалось влияние на течение головной боли различных методов лечения акромегалии. Терапия акромегалии в настоящее время предполагает три возможных варианта: нейрохирургическое (трансназальная транссфеноидальная аденомэктомия), медикаментозное лечение (аналоги соматостатина, антагонисты рецепторов гормона роста и агонисты дофамина), а также лучевая терапия. Целями при лечении акромегалии являются контроль гиперсекреции гормона роста и ИФР-1, удаление опухоли или контроль ее роста, облегчение симптомов, улучшение качества жизни и снижение смертности [41].

## Хирургическое лечение

Хотя головная боль при акромегалии является достаточно значимым клиническим проявлением, в настоящее время в литературе имеется мало достоверных исследований, исходя из которых можно было бы делать окончательные выводы о влиянии нейрохирургического лечения на головную боль [5][11]. Кроме того, после трансназальной транссфеноидальной аденомэктомии могут развиваться такие осложнения, как послеоперационный гипопитуитаризм (вторичный гипотиреоз, вторичный гипокортицизм, вторичный гипогонадизм, центральный несахарный диабет), назальная ликворея, менингит, синдром неадекватной секреции антидиуретического гормона [6]. Тем не менее в некоторых старых проспективных исследованиях было показано, что у многих пациентов с аденомами гипофиза наблюдалось уменьшение интенсивности головных болей, а также частоты возникновения ее приступов после оперативного лечения [5][9][15][39]. В клиническом случае, представленном Rocha et al., также было продемонстрировано исчезновение SUNCT-синдрома после проведенного хирургического удаления гормонально-неактивной аденомы [42]. Напротив, в исследовании Andersson et al. приводятся результаты, в которых отмечен неполный эффект от проведенной операции и даже усиление головных болей [39]. Прогностически благоприятными факторами облегчения головной боли после трансназальной транссфеноидальной аденомэктомии считаются молодой возраст и гормон-продуцирующая микроаденома [15].

## Медикаментозная терапия

Аналоги соматостатина

Соматостатин — пептидный гормон, который секретируется гипоталамусом и желудочно-кишечным трактом. Помимо подавления секреции гипофизом соматотропного гормона, он может ингибировать секрецию субстанции P, CGRP и VIP, которые, как уже было указано выше, могут участвовать в механизмах возникновения головной боли [5]. Двумя наиболее часто используемыми препаратами при лечении акромегалии являются аналоги соматостатина первого поколения (АСС1) — октреотид и ланреотид [41]. В проводимых исследованиях четкой корреляции между наличием головной боли при акромегалии и уровнями сывороточных ИФР-1 и СТГ, а также размерами аденомы гипофиза выявлено не было. Головные боли могут как купироваться, несмотря на отсутствие биохимического контроля заболевания, так и сохраняться после подавления ИФР-1 и СТГ при использовании аналогов соматостатина [43].

В настоящее время известно шесть типов рецепторов соматостатина (SSTR1, SSTR2A, SSTR2B, SSTR3, SSTR4, SSTR5) [5][20][27], которые разделяют на две группы, ингибирующих высвобождение соматотропина 1 и 2 типа: SRIF1 и SRIF2. Рецепторы соматостатина SSTR2, SSTR3 И SSTR5 относятся к группе SRIF1. Cоматостатин и его аналоги через рецепторы данной группы подавляют секрецию гормона роста, а также оказывают антимитотическое действие. В группу SRIF2 включают рецепторы SSTR1 и SSTR4, которые, как предполагается, могут оказывать противовоспалительное и анальгетическое действие [44]. В эндотелии кровеносных сосудов человека экспрессируются рецепторы соматостатина SSTR1 и SSTR4 [45]. По данным литературы, соматостатин, действуя на данные рецепторы, может способствовать вазоконстрикции и, подобно агонистам 5-НТ1 серотониновых рецепторов (триптанам), уменьшать за счет этого головную боль [43].

В старых исследованиях было выяснено, что соматотропиномы активно экспрессируют рецепторы типов SSTR2 и SSTR5, в меньшей мере — SSTR1 и SSTR3 [18][46]. Анальгетические свойства АСС1 могут объясняться высоким сродством к SSTR2 [18], так как данный рецептор, широко представленный в спинномозговом ядре тройничного нерва и периакведуктальном сером веществе [5], играет определенную роль в ноцицепции. Вводимые извне соматостатин и его аналоги в исследованиях приводили к угнетению активности тройничного нерва, что в свою очередь способствовало уменьшению острой кластерной головной боли [47]. В проведенных многоцентровых рандомизированных плацебо-контролируемых испытаниях было обнаружено, что октреотид короткого действия при подкожном введении облегчал головную боль примерно у 80% пациентов с акромегалией. Небольшое обсервационное исследование Kaniuka-Jakubowska et al. (n=18), в котором были отобраны пациенты с акромегалией и головной болью, не имеющие резистентности к АСС1, показало, что использование октреотида пролонгированного действия в комбинации с октреотидом короткого действия уменьшало количество инъекций последнего, что говорит о лучшем контроле головной боли [43]. С рецепторами соматостатина типа SSTR3 и SSTR5 октреотид и ланреотид связываются в меньшей степени [46].

В литературе также имеются данные о применении соматостатина и АСС1 у пациентов без акромегалии с целью обезболивания болей различных локализаций. Так, их применяли для уменьшения боли при онкологических заболеваниях головы и шеи, поджелудочной железы, при метастазах карциноида в костную ткань, при хирургических вмешательствах на органах брюшной полости [43]. Matharu et al. было проведено плацебо-контролируемое исследование, которое показало эффективность октреотида при кластерной головной боли у пациентов без акромегалии. Авторы приходят к выводу, что данный препарат может применяться у пациентов, у которых отсутствует анальгезирующий эффект при приеме триптанов или у пациентов с их непереносимостью [47].

Тем не менее из-за возможного риска развития зависимости рутинное использование аналогов соматостатина исключительно для облегчения головной боли не рекомендуется. Существуют данные о том, что рецепторы соматостатина могут гетеродимеризоваться с опиатными рецепторами, что может привести к усилению головных болей и тахифилаксии при частом использовании этих препаратов [18][48].

У некоторой части пациентов с акромегалией имеет место частичная или полная резистентность к аналогам соматостатина первого поколения [41]. Было показано, что в таких случаях хороший обезболивающий эффект может быть оказан пасиреотидом (не зарегистрирован для лечения акромегалии в РФ) [46] — аналогом соматостатина второго поколения, который имеет высокий аффинитет к четырем типам рецепторов соматостатина (SSTR1, SSTR2, SSTR3 И SSTR5) [44]. Резистентность к АСС1 в литературе связывается с экспрессией аденомой усеченной формы рецептора SSTR5, низкой экспрессией SSTR2, изначально высокими концентрациями гормона роста, высокими значениями Ki-67, редкогранулированным типом соматотропином, низкими уровнями Е-кадгерина в цитоплазме опухолевых клеток и белка, ингибирующего Raf-киназу, наличием мутации в гене AIP, гиперинтенсивным сигналом на Т2-взвешенных изображениях при МРТ гипофиза, молодым возрастом пациентов и мужским полом. Тем не менее влияние данных факторов на отсутствие или недостаточность эффекта терапии октреотидом и ланреотидом остается все еще неясным и требует дальнейшего изучения [46][49].

Агонисты дофамина

Агонисты дофамина (бромокриптин, каберголин) при акромегалии могут использоваться при сочетанной гиперсекреции аденомой СТГ и пролактина или в случае наличия у пациента с акромегалией резистентности к АСС1 [50]. В разных исследованиях было показано, что агонисты дофамина могут способствовать как облегчению головной боли при аденомах гипофиза, так и усиливать ее [18][51][52]. При лечении пролактином агонистами дофамина ремиссия головной боли достигается чаще, чем при лечении акромегалии, вероятно, из-за уменьшения размеров опухоли [5].

Антагонисты рецепторов гормона роста

Пэгвисомант — аналог человеческого гормона роста, связываясь с его рецепторами на поверхности клеток периферических тканей, блокирует эти рецепторы. В частности, способствует значительному уменьшению продукции ИФР-1, блокируя рецепторы СТГ на мембране гепатоцитов. Данный препарат назначается пациентам с частичной или полной резистентностью к АСС1. Однако ввиду отсутствия достаточного клинического опыта, пэгвисомант не показан для лечения головной боли как отдельного симптома у пациентов с акромегалией. Имеются данные о том, что в течение первого года на фоне лечения пэгвисомантом у некоторых пациентов отмечался рост соматотропиномы, что, возможно, способствует усилению головной боли. Рекомендуется периодически выполнять повторные МРТ-исследования для оценки размера опухоли [49][50]. Кроме этого, согласно утвержденной инструкции по применению препарата, головная боль является частым побочным эффектом при инициации лечения пэгвисомантом [53].

## Лучевая терапия

Лучевая терапия является альтернативным методом лечения, если хирургическое вмешательство и/или медикаментозное лечение противопоказано или было малоэффективным, например, в связи с инвазией аденомы гипофиза в кавернозный синус, когда невозможно выполнить ее полное удаление. Однако данный метод лечения низкоэффективен в облегчении головных болей, так как результат может проявляться в течение 5–10 лет, а в некоторых случаях даже усугублять симптоматику [5, 15]. В ретроспективном исследовании Losa et al. было отмечено, что у 8 из 83 пациентов с акромегалией (9,6%) возникло временное усиление головной боли в раннем постлучевом периоде [54]. Giustina et al. делают предположение о том, что это может быть связано с компрессией соседних внутричерепных структур отеком остаточной опухолевой ткани в ответ на облучение. Это подтверждается успешным купированием симптомов глюкокортикостероидной (ГКС) терапией в высоких дозах [5].

## Лечение первичных головных болей

Для лечения первичных головных болей при акромегалии в неврологической практике используются стандартные алгоритмы, как для общей популяции. Эффективность неврологических препаратов для лечения головных болей у пациентов с акромегалией мало изучена, мы не нашли данных в литературе.

Для лечения головной боли напряжения, согласно клиническим рекомендациям, используются поведенческая и профилактическая терапии, а также медикаментозная — для купирования приступов: парацетамол в дозе 1000 мг, нестероидные противовоспалительные препараты (НПВП) — ибупрофен 400 мг, кетопрофен 25 мг, ацетилсалициловая кислота 500–1000 мг и др., при этом наименее эффективным в облегчении головной боли считается парацетамол, а ацетилсалициловая кислота обладает меньшим обезболивающим эффектом, чем другие НПВП. Также необходимо помнить о рисках развития осложнений со стороны желудочно-кишечного тракта, таких как НПВП-гастропатия и желудочно-кишечное кровотечение [55].

Пациентам с частыми приступами ГБН может быть предложена профилактическая терапия, которая направлена на уменьшение количества принимаемых обезболивающих препаратов и на улучшение качества жизни и которая предполагает прием антидепрессантов, например, селективных ингибиторов обратного захвата серотонина и норадреналина (амитриптилин 50–150 мг в сутки, венлафаксин 150 мг в сутки) [55].

Для терапии ГБН не используются триптаны, опиоиды, так как при их приеме высок риск развития ЛИГБ. При наличии выраженной дисфункции перикраниальных мышц дополнительно могут быть использованы миорелаксанты [55].

Современное лечение мигрени, как и лечение ГБН, включает поведенческую терапию, купирование приступов и профилактическую терапию. Для купирования легких и среднетяжелых приступов мигрени применяются НПВП (ацетилсалициловая кислота 1000 мг, ибупрофен 200–800 мг и др.) и парацетамол 1000 мг, а также противорвотные средства (метоклопрамид, домперидон). При тяжелых приступах мигренозной боли используются агонисты 5-НТ1 серотониновых рецепторов, или триптаны (суматриптан, золмитриптан, элетриптан) [56][57].

С целью профилактики приступов мигрени пациентам могут назначаться бета-адреноблокаторы (метопролол, пропранолол), противосудорожные препараты (вальпроевая кислота, топирамат, габапентин), антидепрессанты (амитриптилин, венлафаксин), блокаторы рецепторов ангиотензина II (кандесартан) [56].

Помимо этого, в последнее время для облегчения приступов мигрени появились новые препараты: агонисты 5-HT1F рецепторов (дитаны) и антагонисты рецепторов CGRP (гепанты) [57]. Обезболивающий эффектов дитанов связан с торможением высвобождения CGRP из нейронов тройничного ганглия, а гепантов — с блокадой рецепторов CGRP. Данные группы препаратов не обладают сосудосуживающим эффектом и могут быть рекомендованы пациентам, которым противопоказаны триптаны в связи патологией с сердечно-сосудистой системы [58]. Однако данные препараты пока не зарегистрированы в РФ.

Еще одной новой группой препаратов являются моноклональные антитела к рецепторам CGRP (эренумаб) и непосредственно к самому CGRP (фреманезумаб) [57]. Данные препараты хорошо показали себя в профилактике хронической мигрени [58][59], которая, исходя из проведенных исследований, наиболее часто встречается у больных акромегалией [7–10]. Также только при хронической мигрени используются инъекции ботулотоксина типа А [56][57].

Для лечения острых приступов кластерной головной боли используются подкожное или интраназальное введение триптанов, а также ингаляции 100% кислорода. Несмотря на обилие препаратов, используемых в лечении и профилактике других видов головной боли, при кластерной головной боли наилучшую эффективность показали верапамил и препараты лития. Имеются данные по применению топирамата, габапентина, вальпроевой кислоты и мелатонина для профилактики кластерной головной боли, а также гальканезумаба (моноклональное антитело к CGRP) при эпизодической кластерной головной боли. Тем не менее проводились только открытые исследования, поэтому данные вещества не могут быть одобрены для профилактики кластерной головной боли. Для промежуточной терапии кластерной боли, которая применяется у пациентов в ожидании начала действия профилактического лечения, используются пероральный прием ГКС (преднизолон) или блокада затылочного нерва при помощи ГКС [60][61].

Практикующим врачам необходимо не только помнить, но и сообщать пациентам о том, что чрезмерное употребление препаратов с целью купирования головных болей (НПВП ≥15 дней в месяц, триптаны ≥10 дней в месяц) может быть причиной развития ЛИГБ. Помимо этого, следует минимизировать прием комбинированных препаратов, содержащих кофеин, кодеин, барбитураты, так как они также могут повышать риск развития ЛИГБ [56].

Пациентам также рекомендуется вести дневник головной боли для проведения дифференциальной диагностики между разными формами головной боли при постановке диагноза и для оценки качества проводимого лечения [55].

## Другое

В литературе имеются данные о применении при головной боли, ассоциированной с ВЧГ, с различной эффективностью ингибитора карбоангидразы (ацетазоламид), топирамата, осмотических, тиазидных, петлевых (фуросемид) диуретиков, ГКС (преднизолона, дексаметазона), люмбальной пункции, вентрикулоперитонеального шунтирования, которые использовали с целью снижения внутричерепного давления [12, 20, 21, 62, 63]. Если ВЧГ развилась на фоне лечения СТГ-дефицита препаратами гормона роста, то рекомендуется снижать дозировку или, если есть такая возможность, полностью отменять данные препараты [62].

Даже при достижении ремиссии акромегалии суставные боли при патологии ВНЧС, а также головные боли вследствие нее могут сохраняться, так как изменения черт лица и деформации нижней челюсти не исчезают. Боли в таком случает купируются при назначении НПВП, миорелаксантов, антидепрессантов (амитриптилин), анксиолитиков (бензодиазепины), противосудорожных препаратов (диазепам, габапентин), внутрисуставных инъекций ГКС в ВНЧС [30].

## ЗАКЛЮЧЕНИЕ

Пациенты с аденомами гипофиза, секретирующими гормон роста, часто страдают от сильной головной боли, которая приводит не только к снижению качества их жизни, но даже может стать причиной инвалидизации. Патогенетические механизмы этой головной боли остаются недостаточно изученными, хотя, вероятно, представляют собой комбинацию механических, биохимических, сосудистых, психологических и других механизмов, а также индивидуальной предрасположенности пациента к головной боли.

Цефалгический синдром у больных акромегалией может не купироваться полностью даже в случае достижения ремиссии основного заболевания, поэтому не может быть критерием эффективности лечения акромегалии. Лечащий врач должен предупреждать пациентов об этом, чтобы избежать неоправданных ожиданий. В таких случаях пациентов необходимо направлять к неврологу, специализирующемуся на диагностике и лечении головной боли (цефалгологу), или в специализированный центр лечения боли (головной боли).

## ДОПОЛНИТЕЛЬНАЯ ИНФОРМАЦИЯ

Источники финансирования. Работа выполнена по инициативе авторов без привлечения финансирования.

Конфликт интересов. Авторы декларируют отсутствие явных и потенциальных конфликтов интересов, связанных с содержанием настоящей статьи.

Участие авторов. Нуруллина Г.М. — концепция и дизайн статьи, поиск и анализ литературы, написание и редактирование текста; Пушкарев И.Н. — поиск и анализ литературы, написание текста; Пржиялковская Е.Г. — поиск и анализ литературы, редактирование текста. Все авторы одобрили финальную версию статьи перед публикацией, выразили согласие нести ответственность за все аспекты работы, подразумевающую надлежащее изучение и решение вопросов, связанных с точностью или добросовестностью любой части работы.
